# Supplementary figures and images for: Conformity bias in the cultural transmission of music sampling traditions
Source: R Soc Open Sci. 2019 Sep 25;6(9):191149. doi: 10.1098/rsos.191149 (PMC6774939; doi:10.1098/rsos.191149)

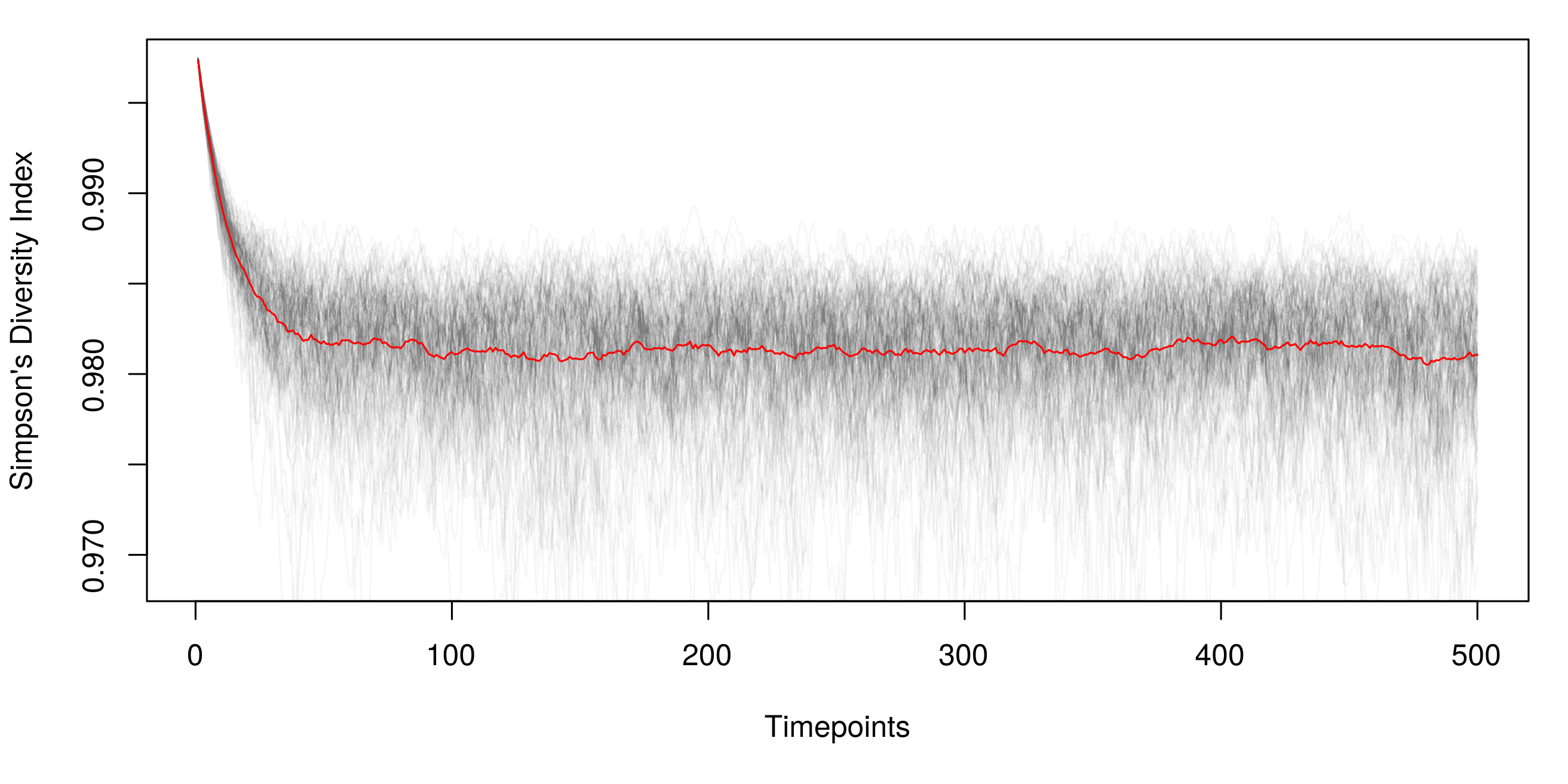

Supplement: Figure S1 [file rsos191149supp1.png]

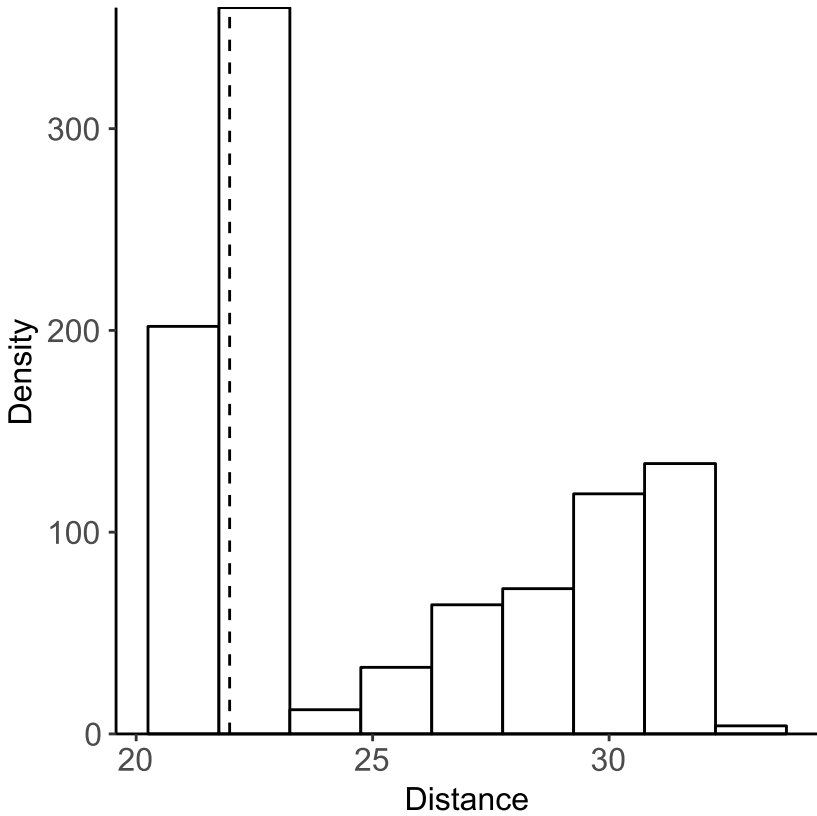

Supplement: Figure S2 [file rsos191149supp2.pdf]

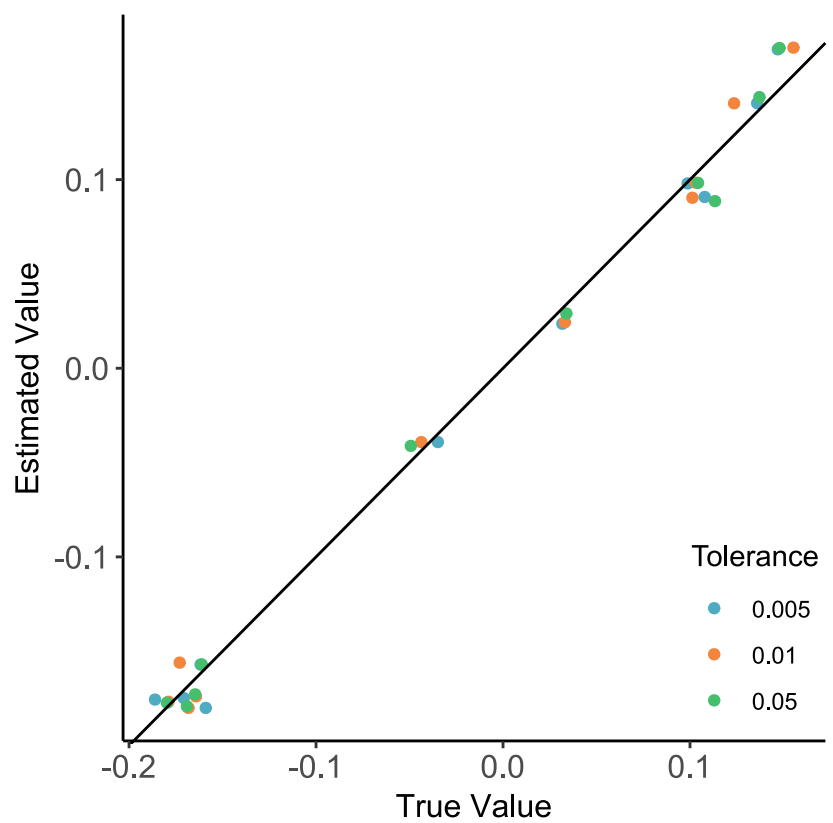

Supplement: Figure S3 [file rsos191149supp3.pdf]

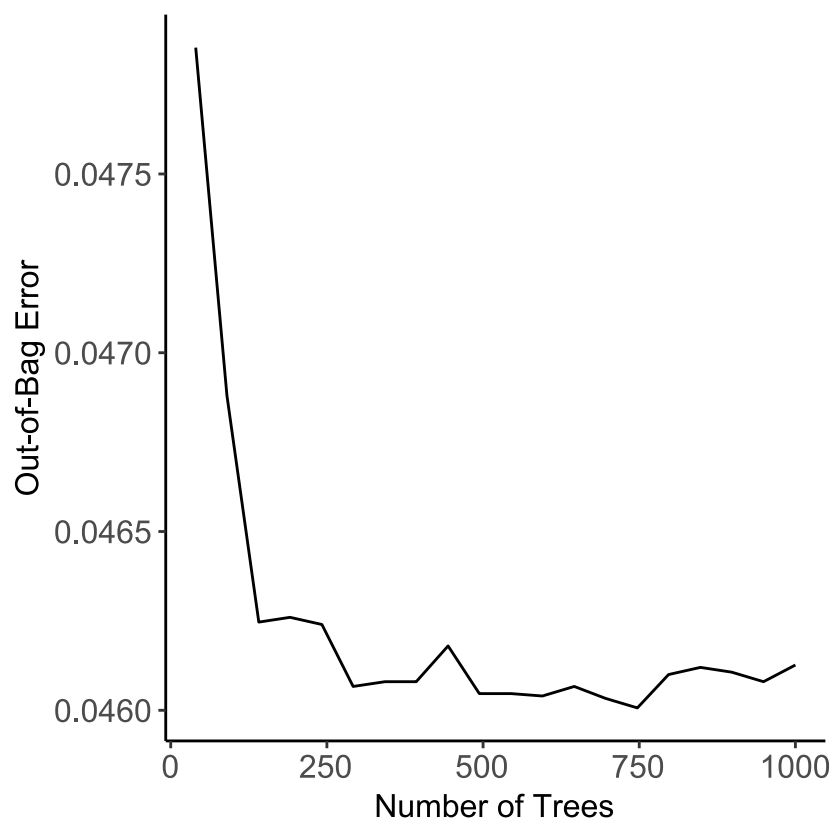

Supplement: Figure S4 [file rsos191149supp4.pdf]

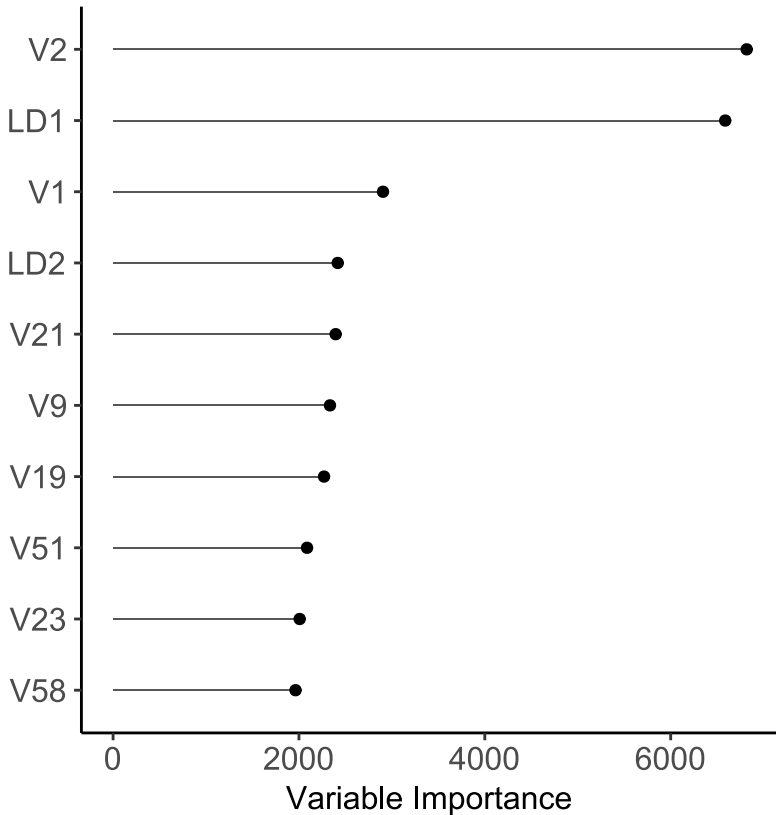

Supplement: Figure S5 [file rsos191149supp5.pdf]

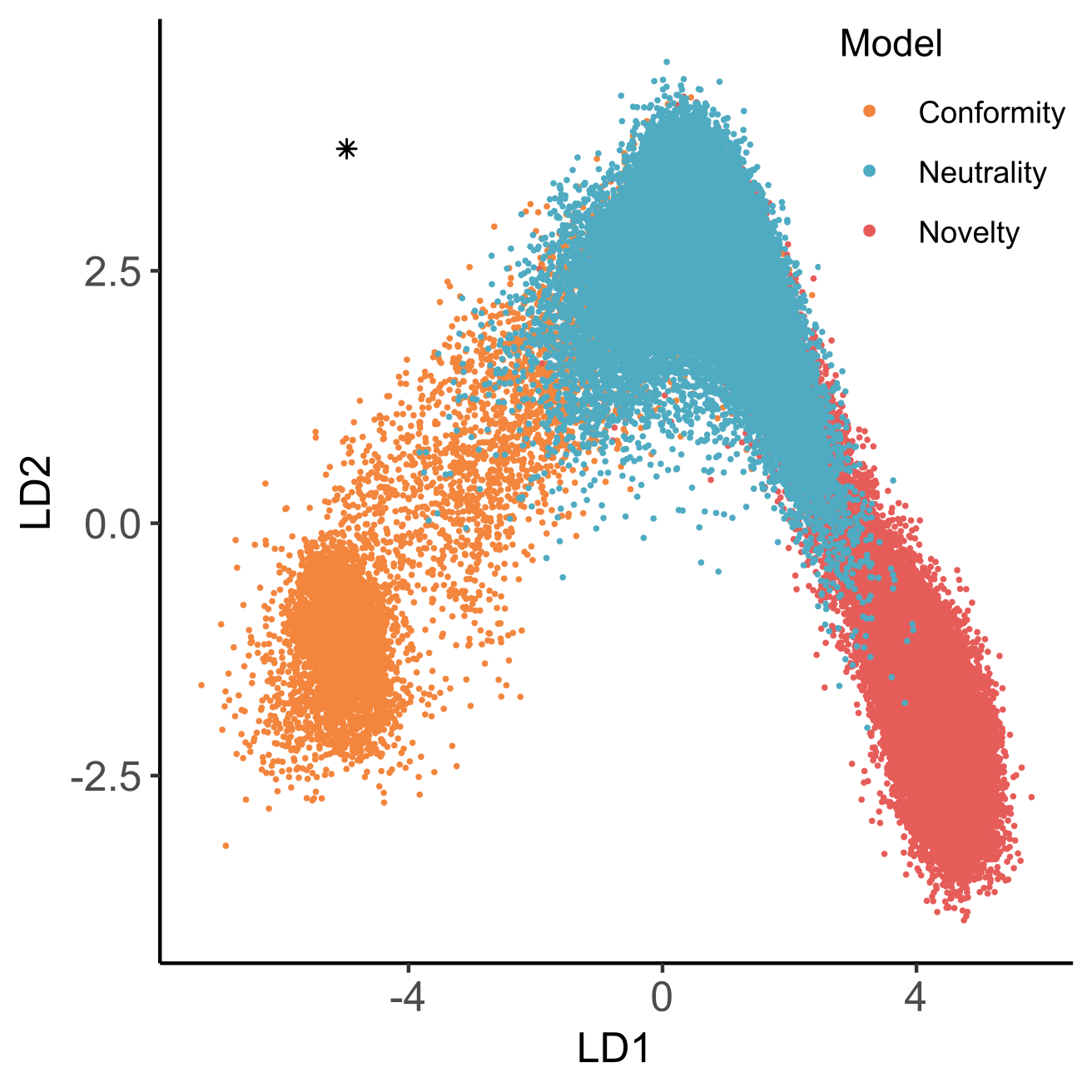

Supplement: Figure S6 [file rsos191149supp6.png]
